# European study on the attitude of psychiatrists towards their patients

**DOI:** 10.1192/j.eurpsy.2021.340

**Published:** 2021-08-13

**Authors:** D. Ori, P. Szocsics, T. Molnar, K. Guevara, L. Bankovska-Motlova, I. Ivanovic, E.A. Carbone, K. Kotsis, E. Dashi, G. Ahmadova, A. Panayi, H. Yilmaz Kafali, I.M. Klinkby, K. Bruna, M. Vircik, M. Wallies, H. Kisand, A. Hargi, A. Mirkovic, P. Rus Prelog, C. Cabaços, A.T. Pereira, S. Boivin, V. Angyal, N. Grinko, G. Grech, F. Schuster, M. Valdivielso, S. Raaj, J. Maslak, S. Mörkl, R. Strumila, N. Nechepurenko, O. Kazakova, S. Kakar, M. Abdulhakim, S. Matheiken, V. Oanca, I. Salopek, G. Kalpak, Z. Gyorffy

**Affiliations:** 1 Acute Ward, Vadaskert Child and Adolescent Psychiatric Hospital, Budapest, Hungary; 2 Institute Of Experimental Medicine, Institute of Experimental Medicine, Budapest, Hungary; 3 Psychiatry, University of Pécs Medical School, Petz Aladár County Hospital, Győr, Hungary; 4 Department Of Psychiatry, Military Medical Academy, Sofia, Bulgaria; 5 Charles University, 3rd Faculty of Medicine, Prague, Czech Republic; 6 Clinic For Psychiatry, Clinical Centre of Montenegro, Podgorica, Montenegro; 7 University Magna Graecia Of Catanzaro, University Magna Graecia of Catanzaro, Catanzaro, Italy; 8 Department Of Psychiatry, University of Ioannina, Ionannina, Greece; 9 Xhavit Gjata Hospital, Xhavit Gjata Hospital, Tirane, Albania; 10 City Hospital N15, City Hospital N15, Baku, Azerbaijan; 11 Private Practice, private practice, Nicosia, Cyprus; 12 Ankara City Hospital Bilkent, Ankara City Hospital Bilkent, Ankara, Turkey; 13 Child And Adolescent Psychiatric Department, Child and Adolescent Psychiatric Department, Region of Zealand, Denmark; 14 Psychiatric Hospital Gintermuiza, Psychiatric Hospital GintermuizaPsychiatric Hospital Gintermuiza, Jelgava, Latvia; 15 Psychiatric Hospital Michalovce, Psychiatric Hospital Michalovce, Michalovce, Slovak Republic; 16 Psychiatrische Klinik Clienia Littenheid, Psychiatrische Klinik Clienia Littenheid, Sirnach, Switzerland; 17 University Of Tartu, University of Tartu, Tartu, Estonia; 18 Child And Adolescent Psychiatry, Children’s Hospital Ljubljana, Ljubljana, Slovenia; 19 University Psychiatric Clinic Ljubljana, Centre for Clinical Psychiatry, Ljubljana, Slovenia; 20 Department Of Psychological Medicine, Faculty of Medicine, University of Coimbra, Coimbra, Portugal; 21 Institute Of Psychological Medicine, Faculty Of Medicine, University of Coimbra, coimbra, Portugal; 22 Epsm Étienne Gourmelen, EPSM Étienne Gourmelen, Quimper, France; 23 Child And Adolescent Psychiatric Clinic, Child and Adolescent Psychiatric Clinic, Jönköping, Sweden; 24 Chernivtsi Reginal Mental Hospital, Chernivtsi Reginal Mental Hospital, Chernivtsi, Ukraine; 25 Psychiatry, Mount Carmel Hospital, Attard, Malta; 26 Klinikum Rechts Der Isar, Technischen Universität München, München, Germany; 27 University Of Navarra Clinic, University of Navarra Clinic, Pamplona, Spain; 28 Department Of Liasion Psychiatry, Mater University Hospital, Dublin, Ireland; 29 Institute For Mental Health, Institute for Mental Health, Belgrade, Serbia; 30 Department Of Psychiatry And Psychotherapeutic Medicine, Medical University of Graz, Graz, Austria; 31 Medicine Faculty, Vilnius University, Vilnius, Lithuania; 32 The Serbsky State Scientific Center For Social And Forensic Psychiatry, The Serbsky State Scientific Center for Social and Forensic Psychiatry, Moscow, Russian Federation; 33 Psychiatric Clinic Of Minsk City, Psychiatric Clinic of Minsk City, Minsk, Belarus; 34 Erasmus University In Rotterdam, Erasmus University in Rotterdam, Rotterdam, Netherlands; 35 Department Of Psychiatry, Vrije Universiteit Brussel, Brussels, Belgium; 36 Department Of Psychiatry, Pennine Care NHS Foundation Trust, Oldham, United Kingdom; 37 Child And Adolescent Psychiatry Clinic, SCUC, Cluj-Napoca, Romania; 38 General Hospital Karlovac, General Hospital Karlovac, Karlovac, Croatia; 39 University Clinic Of Psychiatry, University Clinic of Psychiatry, Skopje, North Macedonia; 40 Institute Of Behavioural Sciences, Semmelweis University, Budapest, Hungary

**Keywords:** Stigma, attitude towards patients, mental health related stigma, psychiatrists

## Abstract

**Introduction:**

Many people think that people with mental disorders might be dangerous or unpredictable. These patients face various sources of disadvantages and experience discrimination in job interviews, in education, and housing. Mental health-related stigma occurs not only within the public community, it is a growing issue among professionals as well. Our study is the first that investigates the stigmatising attitude of psychiatrists across Europe.

**Objectives:**

We designed a cross-sectional, observational, multi-centre, international study of 33 European countries to investigate the attitude towards patients among medical specialists and trainees in the field of general adult and child and adolescent psychiatry.

**Methods:**

An internet-based, anonymous survey will measure the stigmatising attitude by using the local version of the Opening Minds Stigma Scale for Health Care Providers. Data gathering started in July this year and will continue until December 2020.

**Results:**

This study will be the first to describe the stigmatising attitude of psychiatric practitioners across Europe from their perspectives.

**Conclusions:**

The study will contribute to knowledge of gaps in stigmatising attitude towards people with mental health problems and will provide with new directions in anti-stigma interventions.

**Disclosure:**

No significant relationships.

